# Study of a novel Ca/P/S-based cement implanted in the osteoporotic goat spine and its clinical application in preventing adjacent vertebral fractures

**DOI:** 10.3389/fbioe.2026.1784212

**Published:** 2026-05-29

**Authors:** Sheng-Min Lan, Bing-Chen Yang, Chien-Ping Ju, Jiin-Huey Chern Lin

**Affiliations:** 1 National Cheng-Kung University Hospital, College of Medicine, National Cheng Kung University, Tainan, Taiwan; 2 Department of Orthopedics, National Cheng-Kung University Medical College and Hospital Dou-Liou Branch, Yunlin, Taiwan; 3 Joy Medical Devices Corp., Kaohsiung, Taiwan; 4 Center for Biomaterials Research, National Cheng Kung University, Tainan, Taiwan; 5 Department of Materials Science and Engineering, National Cheng Kung University, Tainan, Taiwan

**Keywords:** adjacent vertebral fracture, bone substitute, Ca-based cement, clinical application, osteoporotic goat, spine, vertebroplasty

## Abstract

Osteoporotic vertebral compression fracture (OVCF) has been increasing over the last decades. In addition to primary OVCF, the adjacent vertebral fracture (AVF) is a frequent serious complication in OVCF patients receiving percutaneous vertebroplasty or kyphoplasty, resulting in poor long-term outcome and recurrent symptoms. The aim of the present study is to investigate the bone regeneration behavior of a novel Ca/P/S-based resorbable cement (Ezechbone® Cement CBC-400) implanted into surgically created vertebral defects of ovariectomized (OVX) goats. Furthermore, the present study provides a preliminary clinical result of minimally invasive injection of this cement into the osteoporotic vertebral bodies adjacent to polymethyl methacrylate (PMMA)-filled primary OVCF bodies as a preventive measure. After implantation, a new bone network developed within the CBC-400 cement, and the cement residues were intimately integrated into the surrounding newly formed bone structures without interposition of fibrous tissues. The newly formed trabecular networks in the OVX_CBC goat were much denser and thicker than the native networks in the OVX goat and those even in the healthy control goat. Under polarized light, a much more randomly oriented, smaller-sized, and more vibrant lamellar bone morphology was observed in the OVX_CBC goat compared to that in the OVX goat, indicating an excellent bone-regenerative efficacy of CBC-400 cement. The treatment results of 10 consecutive clinical OVCF cases, in which their adjacent vertebral bodies preventively augmented with CBC-400, showed that even at an average age of almost 80 years, with half of the patients having prior osteoporotic vertebral fractures and all having multiple comorbidities, none of these patients developed any new AVF in the following 3 years. Prophylactically augmenting adjacent osteoporotic vertebrae with Ezechbone® Cement CBC-400 in combination with conventional PMMA vertebroplasty for OVCF has demonstrated a high potential for effectively reducing the risk of future adjacent vertebral fractures.

## 1 Introduction

Osteoporosis has become a global health issue today. It is estimated that, worldwide, approximately one in five men and one in three women older than 50 years could, in their lifetime, suffer from one of the osteoporosis-related fractures (Morin et al., 2025). Among these fractures, osteoporotic vertebral compression fractures (OVCFs) have been increasing over the last decades, especially among elderly patients. OVCF commonly leads to anterior vertebral height loss causing spinal deformities, reduced pulmonary function, and even mortality. OVCF can also prolong hospitalization, increase morbidity, and affect quality of life. The osteoporosis-induced thinning of the trabecular structure has been considered a natural consequence of aging, as well as a sign of impaired osteoblast function (Lips et al., 1978; Darby and Meunier, 1981). Such changes in the trabecular structure could compromise bone strength and increase fracture incidence (Eastell, 2017). Similar functional impairment of osteoblasts was also found in osteoporotic patients (Darby and Meunier, 1981).

The standard treatments for OVCF include bed rest, analgesia, bracing, external fixation, rehabilitation, antiosteoporotic medicine, and surgical therapy. Antiosteoporotic medicine consisting of antiresorptive agents that suppress osteoclastic activity and anabolic agents that enhance osteoblastic activity has been developed. The antiresorptive agents include estrogen, selective estrogen receptor modulator (SERM), calcitonin, bisphosphonates, and denosumab. Certain anti-inflammatory drugs and analgesics may cause intolerable side effects, especially in elderly patients. Classical instrumented fusion surgery cannot guarantee success due to the poor quality of osteoporotic bone. A failed surgery would lead to persistent back pain, limited functions, and neurological symptoms.

As minimally invasive spinal surgery techniques emerged in the past few decades, the acute painful vertebral compression fracture could be treated through a percutaneous procedure, such as percutaneous vertebroplasty (PVP) or percutaneous kyphoplasty (PKP). These minimally invasive procedures involve injecting a polymethyl methacrylate (PMMA) polymeric cement into the fractured vertebra to provide an immediate load-bearing capacity, stabilize the fracture site, and relieve the patient’s pain.

In addition to primary OVCF, the adjacent vertebral fracture (AVF) is a frequent and serious complication in OVCF patients receiving PVP or PKP, resulting in poor long-term outcome and recurrence of pain-related symptoms. Uppin et al. (2003) reviewed 177 osteoporotic patients treated with PMMA vertebroplasty and found that 12.4% patients had developed new fractures, of which 67% fractures occurred adjacent to the PMMA-treated vertebral bodies. From 12 review studies on balloon PKP (BKP) using PMMA, Hulme et al. (2006) reported that 766 patients had 115 new fractures, of which 66% were located at an adjacent level. Even in an average follow-up as short as 1 month, Harrop et al. (2004) found that 26 (22.6%) patients developed 34 (15.1%) subsequent fractures out of the 225 vertebrae in 115 patients treated with PMMA-BKP. The subsequent fracture incidence was 11.25% in patients with primary osteoporosis and 48.6% with steroid therapy-related secondary osteoporosis. From 369 patients undergoing percutaneous vertebroplasty and being followed up for over 1 year, Kim et al. (2011) found 51.9% suffering from subsequent AVF. The study also reported an intradiscal cement leakage rate of 33.3% in their fracture group, with 66.7% in the fracture group having a pre-existing fracture. Wang et al. (2018) reported that new adjacent fracture was one major postoperative complication of PVP using PMMA. In this report, the clinical incidence of new adjacent fractures was 12.4%–27.7%. In most cases, additional PVP surgeries were required on these fractured segments, inflicting additional pain and financial burden on the patients.

In a relatively recent study, Park and Park (2021) reported that new vertebral fractures occurred in 47 (22.9%) of 205 patients who received vertebroplasty for OVCF and were followed up for at least 1 year. Of these, AVF occurred in 21 patients (10.2%), while remote vertebral fractures (RVFs) occurred in 26 patients (12.7%). The onset time of AVF, quite differently, was far shorter (6.2 ± 1.8 months) than that of RVF (15.2 ± 1.8 months) after vertebroplasty. The univariate analysis of the study indicated that the risk factors of AVF included severe osteoporosis (T-score < −3.0), vertebroplasty in the thoracolumbar junction, sagittal imbalance, and segmental kyphosis angle >15°. Mao et al. (2021) reviewed the risk factors for secondary fractures to percutaneous vertebroplasty for OVCF involving a total of 1,882 patients, of which 340 (18%) were diagnosed as secondary fractures after percutaneous vertebroplasty. It has been concluded in the study that the risk factors included additional history of fracture, old age, low bone mineral density (BMD), PMMA leakage, intravertebral fracture clefts, and scoliosis.

A network meta-analysis comparing PVP and PKP involving 23 randomized controlled trials with a total of 2,838 patients was conducted by Essibayi et al. (2024). The results showed comparable risks of adjacent fractures between vertebroplasty, kyphoplasty, and natural history after OVCF, with a mean follow-up of 21.2 months, suggesting that these interventional treatments did not significantly increase or decrease the risk of adjacent fractures. In a prospective cohort study on 40 OVCF patients with an average age of 56 ± 8 years, Jindal et al. (2023) also reported that PVP required shorter intraoperative time and was more economical than BKP, while the final radiological, clinical, and functional outcome and overall complications of the two groups were similar. In other words, BKP provided no added benefit over percutaneous vertebroplasty.

Prophylactic vertebroplasty using PMMA, i.e., prophylactically injecting PMMA cement into a non-fractured vertebra during the procedure of PVP for compression fracture, has also been explored. In a retrospective study including 116 OVCF patients undergoing prophylactic PVP using PMMA, Kamano et al. (2011) found that, within 3 months following the procedure, subsequent fractures occurred in 26 vertebrae in 21 patients (18.1%). Within 12 months, subsequent fractures occurred in 36 vertebrae in 28 patients (24.1%). In a relatively recent study, Tao et al. (2022) investigated the potential predictor of AVF following PKP using PMMA. Their clinical data from 445 OVCF patients receiving PKP were reviewed. The authors suggested that BMD, kyphosis angle, cement distribution, and disc degeneration were among the major factors and that a prophylactic treatment might be helpful for reducing the occurrence of AVF. The majority of the review articles showed that AVF occurrence rates are indeed quite high even within a relatively short time post-PVP or -PKP surgery using PMMA.

It is well known that the healing process of a fractured osteoporotic bone can be significantly impaired, and an ovariectomy (OVX) procedure can effectively induce osteoporosis. However, exactly how osteoporosis would impair the bone healing process may not be straightforward. van Houdt C. I. et al. (2018) reported that osteoporosis may alter the implant resorption rate in different ways, making the result rather difficult to predict. Walsh et al. (1997) reported impaired fracture healing in OVX-induced osteoporotic rats. Kubo et al. (1999) histologically observed some osteoporotic features at the fracture site accompanied with a decreased BMD in 12 weeks post-OVX rats. Meyer Jr. et al. (2001) observed a significantly decreased bending strength in OVX rats. Lill et al. (2003) reported delayed fracture healing in osteoporotic sheep tibiae. From their histological, biomechanical, and radiological results, McCann et al. (2008) suggested that OVX caused delayed fracture healing. Cesnjaj et al. (1991) implanted demineralized bone matrix (DBM) intramuscularly into both normal and OVX rats and showed that the DBM taken from normal rats led to good bone formation in both normal rats and the OVX rats. The DBM taken from OVX rats, however, did not improve the osteogenesis process even in normal rats. This result suggested that the OVX-induced changes occurred not only in the native bone but also in the newly regenerated bone. In other words, the newly formed bone in an osteoporotic bone defect could still be osteoporotic.

The impaired bone healing process would supposedly increase the demands of fixation; however, the risk of fixation failure often increases due to the porous structure and low strength in osteoporotic bone. Under this situation, the application of bone graft is often suggested. Autograft has widely been used to repair bone defects. However, its limited availability, additional surgical procedures, morbidity, and risk of fracture in harvesting sites are among some major concerns. Alternative bone healing-enhancing materials include allograft, xenograft, and synthetic materials. The use of synthetic bone grafts would, by nature, abolish various risks derived from allografts or xenografts, such as disease transmission. Calcium phosphate and calcium sulfate are two popularly used synthetic bone substitute materials. Both materials are reported to be capable of stimulating osteogenesis under normal bone metabolism condition (Decker et al., 1995; Ricci et al., 2005).

A synthetic, 100% inorganic, highly porous, and fast resorbable Ca/P/S-based granular material (Ezechbone® Granule CBS-400, Joy Medical Devices Corp., Kaohsiung, Taiwan) has been used in an earlier study (Yang et al., 2020) to fill surgically created vertebral defects of osteoporotic goats. The results of the toluidine blue (TB)-stained histological examination revealed a newly formed trabecular bone network within the defect of the CBS-400-implanted goat. This new trabecular bone network appeared much denser than that in the OVX goat and even denser than that in the healthy control goat. With this encouraging result, we aim to investigate the bone regeneration behavior of a novel Ca/P/S-based orthopedic cement (Ezechbone® Cement CBC-400) implanted into surgically created vertebral defects in OVX goats. Furthermore, serving as a proof-of-concept study, the present study provides a preliminary clinical result of minimally invasive injection of this cement in the osteoporotic vertebral bodies adjacent to the PMMA-filled primary OVCF bodies as a preventive measure.

## 2 Materials and methods

### 2.1 Material used for the study

Ezechbone® Cement CBC-400 used for the study is a Ca/P/S-based bone regeneration material with a delicate design in its chemistry, microstructure, and morphology developed by National Cheng-Kung University (NCKU) and Joy Medical Devices (JMD) Corp., an ISO 13485/GMP and Ministry of Economic Affairs (MOEA)-certified Biotech and New Pharmaceutical Company located at South Science Park, Kaohsiung, Taiwan. CBC-400 is an all synthetic, inorganic, and fully resorbable calcium-based cement material without any content from human or an animal source. Without adding any polymeric or other additives, Ezechbone® Cement is nondispersive when contacting blood/body fluid, minimizing the dangerous risk of cement leakage/embolism. After implantation *in vivo*, CBC-400 is always intimately integrated with the surrounding bone and eventually replaced by the natural, newly formed bone substantially without inflammatory or other undesirable tissue reactions. Ezechbone® Cement CBC-400 has been approved for clinical use in orthopedic and dental fields by Taiwan Food and Drug Administration (TFDA), in which selected safety and efficacy data of the material have been published (Chen et al., 2014). The TB-stained histology indicates that the cement residual ratios in 4-week, 12-week, and 26-week samples are approximately 86%, 43%, and 14%, respectively. Furthermore, CBC-400 can be safely and effectively delivered into deep surgical sites using JMD’s proprietary minimally invasive cement delivery system, JBranch™. A supplementary table summarizing some key material properties is presented, including Ca/P/S ratio, setting time, mechanical strength *in vitro*, porosity, and degradation rate in a physiological medium.

### 2.2 Osteoporotic goat spine implantation and histology

The osteoporotic goat implantation for the study was performed with procedures approved by the local ethics committee, and the experiments were conducted in accordance with the National Institutes of Health Guide for the Care and Use of Laboratory Animals. Four goats were used for the study, of which two goats (OVX_VD and OVX_CBC) were used as surgical groups and two other goats (control and OVX) were used as a healthy control group and an osteoporotic control group, respectively. The OVX_VD surgical group represents the animals wherein a bone void was surgically created without implantation, while the OVX_CBC surgical group represents the animals wherein the surgically created bone void was implanted with CBC-400.

One healthy goat was fed and bred normally as a control, while the other three underwent OVX, fed with a specially prepared low calcium diet, and bred away from light to induce osteoporosis (Kubo et al., 1999; Namkung-Matthai et al., 2001; Clements et al., 1987). For 18 months after OVX, bone voids were surgically created in the vertebral bodies of two OVX-induced osteoporotic goats, while the third goat, designated “OVX goat,” was sacrificed at this time point. The surgically created bone void in the vertebral body of one goat was filled with 1.0 mL Ezechbone® Cement CBC-400 (designated “OVX_CBC goat”), while the surgically created void of the second goat was left without implantation (designated “OVX_VD goat”). The critical-size bone void was an elliptical tunnel (approximately 10 mm in depth), with a major axis of 10 mm and minor axis of 8 mm in width (Yang et al., 2020).

Surgeries were conducted under general anesthesia (Zoletil 50, 25 mg/kg, intramuscular injection; Virbac, Carros, France) accompanied with local anesthesia (xylocaine, 0.2 mg/kg, intramuscular injection; AstraZeneca, Cambridge, UK). After shaving and cleaning with 70% v/v ethanol and Betadine® (povidone iodine 10% w/v), a longitudinal, midline incision was made in the dorsal skin over the lumbar vertebra between L1 and L3. The left paraspinal muscles were elevated and retracted using a Taylor retractor. After operation, all ovariectomized goats, including OVX, OVX_VD, and OVX_CBC goats, were all fed with a normal calcium diet. All these ovariectomized goats, along with the healthy control goat, were sacrificed at 24W post-operatively using an overdose of Zoletil (50 mg/kg) to induce deep anesthesia, followed by intravenous administration of potassium chloride (KCl; 2 mEq/kg) using a 3M solution.

**FIGURE 1:**
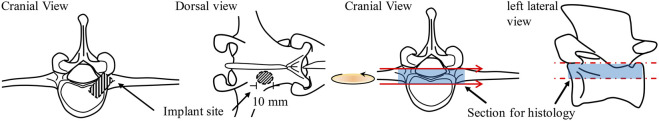
Schematic drawings illustrating the osteoporotic goat implantation site and sample sectioning for histology.

After sacrifice, the lumbar vertebrae were immediately excised with excess tissues removed. From the healthy control goat and the OVX goat without surgical treatment, the L3 vertebral bodies were taken for the study. The vertebral bodies were taken from the sections close to the middle of the implant site, as illustrated in Figure 1. Samples were sectioned into four thick slices using a Buehler low-speed diamond blade (IsoMet Blade, Buehler, Illinois, USA). The high-contrast TB-stained histological micrograph, as shown in a previous study (Yang et al., 2020), revealed the large empty space typically observed in OVX_VD goats, indicating that the size of the surgically created defect was large enough so that it could not be repaired via the normal bone healing mechanism.

Sectioned samples were fixed in 10% w/v neutral buffered formalin (NBF) (pH 7.0) for 3 days, dehydrated in increasing grades of ethanol, and embedded in Buehler EpoxiCure 2 resin. Each embedded sample was sectioned into two parts: one for preparing thin sections for polarized transmitted light microscopy and the other for reflected light microscopy. Transmitted polarized light microscopy was used primarily for the examination of bone–cement interface, bone ingrowth behavior, collagen fiber orientations, etc. The samples were ground and polished through 0.05 μm Al_2_O_3_ powder, glued to slides with resin, and thinned to a final thickness of approximately 100 µm. These thin samples were stained with TB and sealed with Permount (Fisher Scientific, Fair Lawn, NJ, USA). A polarized light microscope (DM2500P, Leica Co., Germany) was used for the study. TB is commonly used in staining undecalcified bone sections (Lowe et al., 2018). Relatively mature lamellar bones are usually aligned in layers, and collagen bundles are usually parallel to each other, causing a birefringence effect that allows identification of their existence under polarized light (Bullough, 2010; Warshaw et al., 2017). In contrast, it is rather difficult to detect collagen fiber orientations using reflection mode. The transmitted polarized light microscopy used in the present study could easily distinguish lamellar bone from woven bone based on their different birefringent effects (Gunson et al., 2015; Ip et al., 2016).

Samples for reflected light examination were prepared using the same procedures as for polarized light examination. To enhance resolution while simultaneously obtaining an overall picture demonstrating trabecular features, a large number of micrographs were taken sequentially and superimposed to form a composite picture covering the entire bone/cement cross-section using Leica Application Suite software.

For histological and histomorphometric evaluation, four slices of samples from each group were examined. To evaluate the histological parameters, specifically bone area fraction and trabecular thickness, of the OVC_IP goat, two distinct regions of interest (ROIs) were analyzed. One was the peri-implant region surrounding the implanted cement, while the other was a distal region representing naive osteoporotic bone. Four sections of the same implantation site of the OVC_IP goat were analyzed. To ensure consistency, the total areas of the two ROIs remained the same across all sections. The methodology for calculating bone area fraction and trabecular thickness followed the protocols previously described in an earlier study (Yang et al., 2020). Trabecular thickness data from multiple sections were visualized using Violin SuperPlots (Kenny and Schoen, 2021) to simultaneously display the distribution of individual measurements and their central tendencies between two ROIs. The quantitative data are presented as means ± standard deviations. Paired Student’s t-test was used to compare the differences in histomorphometry between the two ROIs, wherein significance was considered at *p* < 0.05.

### 2.3 Clinical procedures and follow-ups of AVF preventive treatment

In 2019, 10 consecutive vertebral compression fracture patients with an average age of 79.6 ± 8.87 years/o were enrolled. All patients were female. The average follow-up period was 2.8 years, with the longest follow-up extending up to 5 years. Out of the 10 patients, 5 (50%) had prior osteoporotic vertebral fractures. The underlying diseases of the patients included chronic kidney disease, end-stage renal disease, hyperparathyroidism, bladder cancer, multiple myeloma, chronic heart failure, adrenal or thyroid insufficiency, Parkinson’s disease, and vertebra-basilar insufficiency, among others. In other words, the patients included in this study all have multiple comorbidities. Moreover, the extent of osteoporosis was quite serious, with dual-energy X-ray absorptiometry (DXA) demonstrating an average T-score of −3.84 ± 0.94. Their fractures were all caused by minor trauma, which indicated osteoporotic fractures. When an OVCF was identified in an outpatient or emergency department, conservative treatment was started first. After more than 4 weeks of conservative treatment failed, surgery was conducted.

The surgery involved performing a conventional vertebroplasty by injecting PMMA bone cement into the fractured vertebra to provide immediate load-bearing capacity and to relieve the pain. Moreover, the adjacent vertebrae were augmented with Ezechbone® Cement CBC-400 in an attempt to reduce the risk of future AVF. The CBC-400 cement was delivered trans-pedicularly using a proprietary minimally invasive cement delivery system, JMD CDS-2000. All radiographs were reviewed and interpreted by an orthopedic surgeon with more than 15 years of clinical experience in the field, along with a diagnostic radiologist with more than 20 years of experience. Because this is a retrospective study and the study did not adversely affect the rights and welfare of the patients, the Institutional Review Board at National Cheng Kung University Hospital (NCKUH) approved the study protocol (A-ER-109-031) and agreed to waive the patient’s informed consent for data publication.

Preoperative, postoperative, and 1-year follow-up pain visual analog scale (VAS) scores were recorded for all 10 patients receiving the present AVF-preventive treatment. The quantitative data are presented as means ± standard deviations. The statistical analysis was performed using the Wilcoxon signed-rank test for paired samples as VAS scores were non-parametrically distributed.

## 3 Results

### 3.1 Histology and bone morphology examination

**FIGURE 2:**
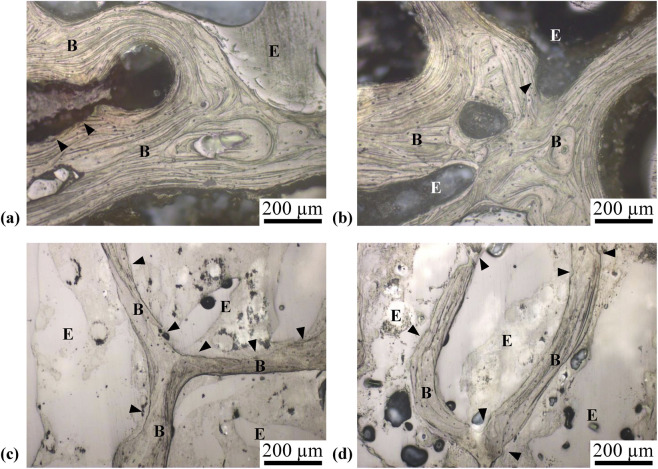
Typical histologic micrographs of the healthy **(a,b)** and ovariectomy (OVX)-induced osteoporotic **(c,d)** goat spine under normal reflected light. B: Bone; E: epoxy. Black arrowheads indicate the etched surface of the bone resorbed by osteoclast. Compared to the healthy control goat, the OVX goat has significant reduction in trabecular thickness, trabecular number, and bone area.

**FIGURE 3:**
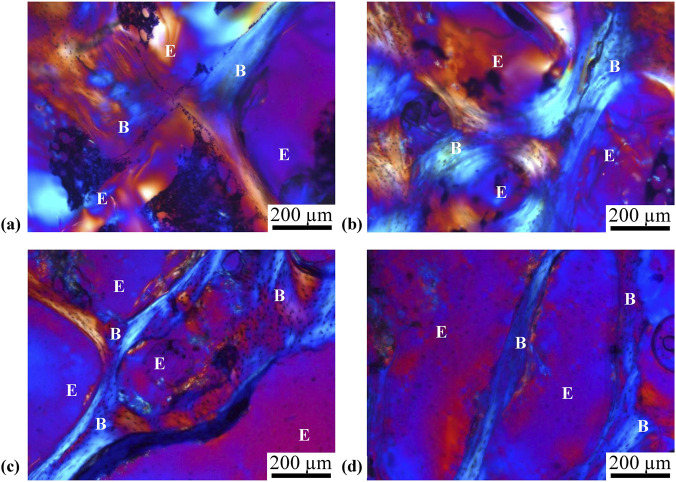
Typical TB-stained histologic micrographs of healthy **(a,b)** and OVX-induced osteoporotic **(c,d)** goat spine under polarized transmitted light. B: bone; E: epoxy. The healthy goat displays a denser and more randomly oriented trabecular feature than the OVX goat.

Typical histological micrographs of healthy control goat and OVX-induced osteoporotic goat spine under normal reflected light are presented in Figure 2a–d, respectively. Figure 2 also reveals that, compared to the healthy control goat, the OVX goat had significant reduction in trabecular thickness, trabecular number, and bone area, accompanied with an increase in inter-trabecular space. TB-stained histological micrographs of healthy and OVX goat spine under polarized transmitted light are shown in Figure 3a–d, respectively. Under polarized light, the blue and orange/red colors represent two perpendicularly oriented fiber orientations, whereas the purple color typically represents a rather randomly orientated region without specific fiber features. It can be observed that, under polarized light, the healthy goat displayed not only a denser but also a more randomly oriented trabecular feature than the OVX goat. These results indicated that the present OVX procedure successfully induced osteoporosis in the experimental female goats.

**FIGURE 4:**
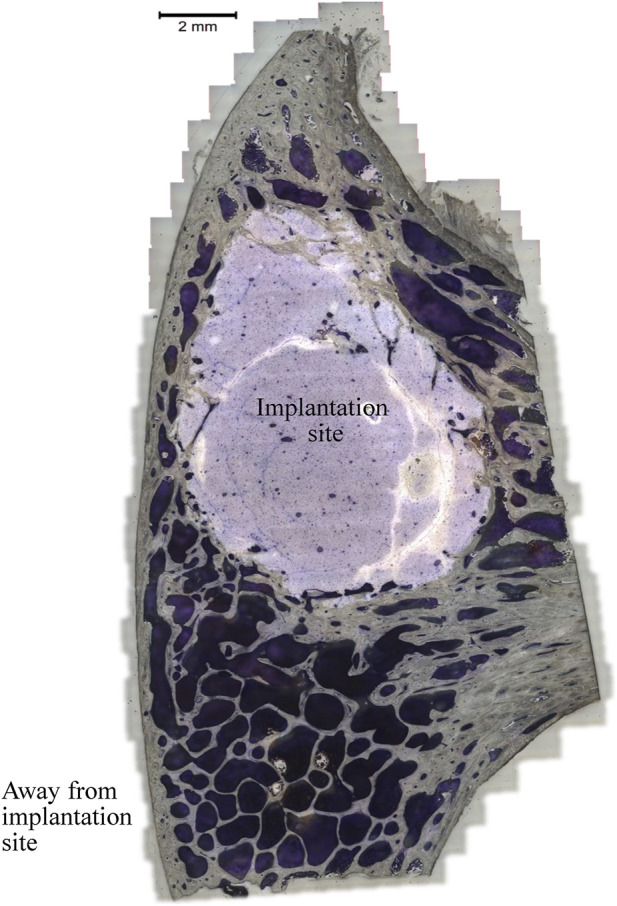
Typical histologic micrographs of the OVX_CBC goat spine at the implantation site and away from the implantation site under normal reflected light. A tight, continuous interface is observed throughout the entire implantation site between CBC-400 implant and the host bone substantially without interposition of fibrous tissues. During the resorption process, the cement is gradually disintegrated into small islands, further enhancing the resorption process.

**FIGURE 5:**
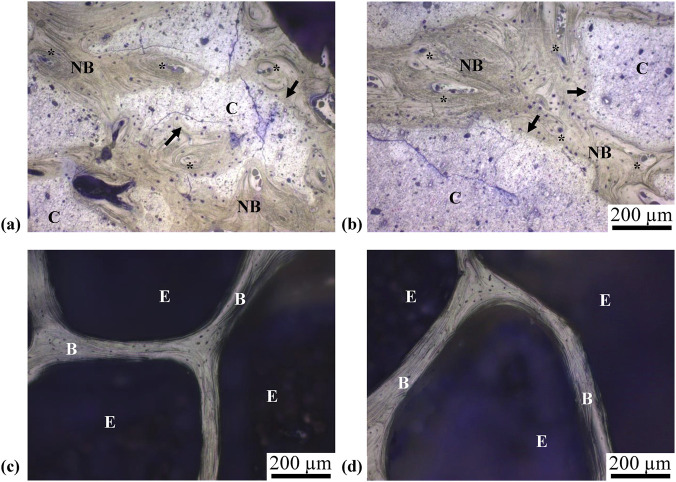
Typical histologic micrographs of the OVX_CBC goat spine at the implantation site **(a,b)** and away from the implantation site **(c,d)** under normal reflected light. B: Bone; E: epoxy; NB, new bone. Asterisks indicate blood vessels. Black arrows indicate the interface between new bone and implanted CBC-400. The trabeculae away from the cement are always much thinner than those near the cement.

Figure 4 represents typical histological examination of OVX_CBC goat spine under normal reflected light. As indicated in the figure, Ezechbone® Cement CBC-400 was nondispersive and well confined within the implanted site after being implanted in the surgically created bony cavities of osteoporotic goat’s vertebral body. A tight, continuous interface was observed throughout the entire implantation site between CBC-400 implant and the host bone substantially without interposition of fibrous tissues. During the resorption process, the cement was gradually disintegrated into small islands, further enhancing the resorption process, as demonstrated in Figure 5a,b. Evidence of resorption, indicated by the etched features on the bone surface (indicated by arrows in Figure 2c,d), was commonly observed in the OVX goat but was hardly found in the healthy control or OVX_CBC goat. Although very few blood vessels were observed in the trabeculae of the control goat and OVX goat, quite a few blood vessels and newly developed trabecular packets surrounding the implanted cement were easily discerned in the trabecular bone structure of the OVX_CBC goat.

**FIGURE 6:**
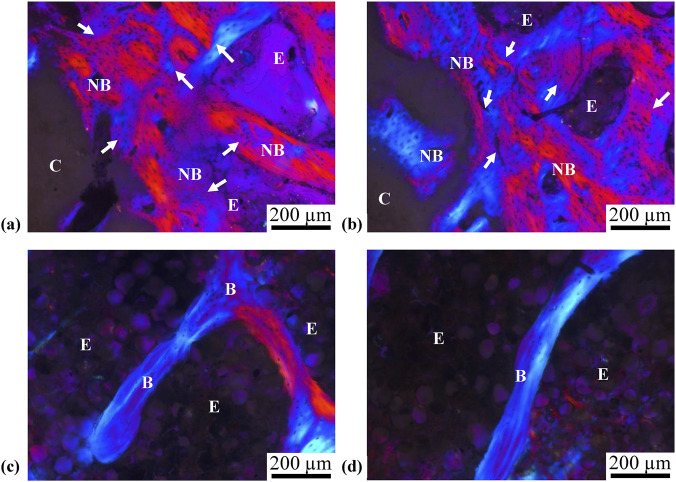
Typical TB-stained histologic micrographs of the OVX_CBC goat spine at the implantation site **(a,b)** and away from the implantation site **(c,d)** under polarized transmitted light. White arrows in **(a)** and **(b)** indicate the transition between two different lamellar orientations reflecting a more randomly oriented and vibrant trabecular morphology.

The efficacy of CBC-400 in inducing new bone formation in the osteoporotic goat spine is further demonstrated in Figures 5, 6. Interestingly, under higher magnification, the thickness and coverage of the newly developed trabecular bone near the implanted cement were far greater than those away from the cement. A seamless bone–implant interface was observed throughout the entire implantation site substantially without interposition of fibrous tissues. A typical example is provided in Figure 5, in which the trabeculae away from the cement were always much thinner than those near the cement. Again, the TB-stained micrographs under polarized transmitted light revealed denser and more randomly oriented trabecular morphology near the cement than that away from the cement (Figure 6). The longer-appearing lamellar bones were observed mostly parallel to the implant surface away from the cement, highly similar to that observed in the OVX goat. On the other hand, numerous dark gray-colored CBC-400 residues were tightly embedded among the surrounding active bones near the cement. Figure 6 also reveals that the area of new bone (blue bands and orange/red bands) near the cement was much smaller-scaled than that away from the cement and comparable to that in the healthy control goat (Figure 3a,b), indicating an excellent bone-regenerating efficacy of Ezechbone® Cement CBC-400.

**FIGURE 7:**
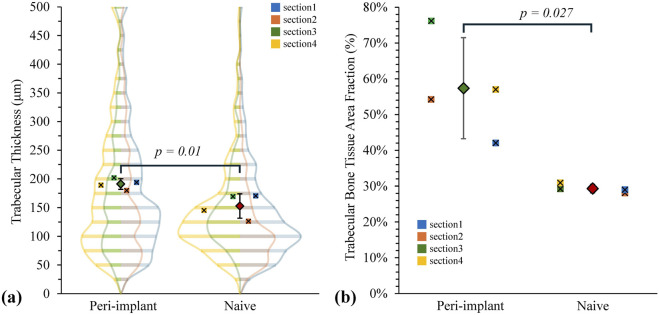
Histomorphometry parameters of the peri-implant and naive regions of the OVX_IP goat. **(a)** Trabecular thickness; **(b)** trabecular bone tissue area fraction. The peri-implant region has higher average trabecular thickness and trabecular bone tissue area ratio than the naive region.

The quantitative data of trabecular thickness and the trabecular bone tissue area ratio of the four sections are presented in Figure 7, in which the squares with crosses represent the average values of each section. The diamonds indicate the means of the average values for the four sections with standard deviations. The violin superplots illustrate the distribution of trabecular thickness, showing that the naive regions exhibit a narrower distribution concentrated toward smaller thicknesses than the peri-implant regions. Figure 7 summarizes the results of histomorphometry of the different ROIs from the OVX_IP goat. As indicated in Figure 7a, the peri-implant region has a higher average trabecular thickness (191 μm) than the naive region (153 μm). The difference is significant (by 24.8% increase, *p = 0.010*). Figure 7b indicates that the average trabecular bone tissue area ratio in the peri-implant region (57.4%) is also much higher than that in the naive region (29.3%), with a significant difference (by 95.9% increase, *p = 0.027*).

### 3.2 Clinical application of Ezechbone® Cement CBC-400 for AVF-preventive treatment

All the aforementioned clinical operations went smoothly, and the patients were fitted with back braces for 2 months after surgery. Each patient was followed up for at least 1 year, with the longest follow-up lasting nearly 6 years. The average follow-up period was 2.83 ± 1.87 years. Six of these ten patients had passed away due to unrelated causes. From the records traced, none of these patients had ever encountered any new fractures in the adjacent vertebrae that were preventively filled with Ezechbone® Cement CBC-400. Based on the results of this study, it can be concluded that preventively strengthening adjacent osteoporotic vertebrae with Ezechbone® Cement CBC-400 in combination with conventional PMMA vertebroplasty for OVCF effectively reduces the risk of future AVF.

**FIGURE 8:**
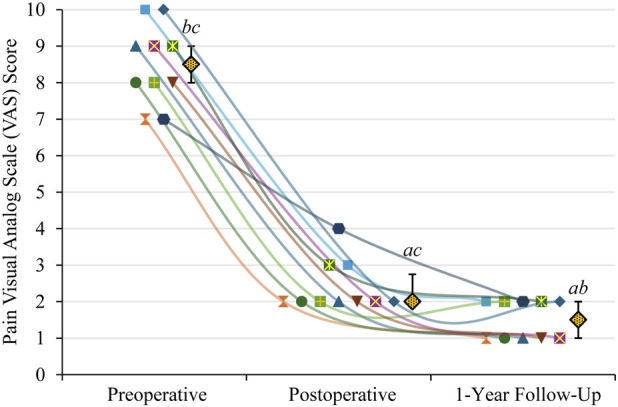
Changes in pain visual analog scale (VAS) scores of the 10 patients with AVF-preventive treatment are presented as median with interquartile range. Symbols *a*, *b*, and *c* indicate that the median value has a significant difference (*p* < 0.05) compared to preoperative, postoperative, and 1-year follow-up time points, respectively.

Changes in VAS scores of the 10 patients with AVF-preventive treatment are presented in Figure 8. Preoperatively, all patients suffered from moderate to severe pain, with VAS scores ranging from 7 to 10 (median [IQR]: 8.5 [8.0–9.0]), indicating that this disease had severely affected the patients' quality of daily life, justifying the indication for surgical intervention. In the immediate postoperative period, pain relief was significant, with VAS scores decreasing to 2–4 (median [IQR]: 2.0 [2.0–2.75]). Although acute discomfort from the surgical site remained, the intensity of pain was all substantially reduced compared to their preoperative baselines. At 1 year postoperatively, all patients achieved excellent pain relief, with VAS scores of either 1 (n = 5) or 2 (n = 5). No patients reported a score greater than 2 at the final follow-up.

Among the 10 cases of this study, four cases with relatively longer follow-up periods (3 years or longer post-operatively) are presented in detail in the following sub-sections.

#### 3.2.1 Case 1

**FIGURE 9:**
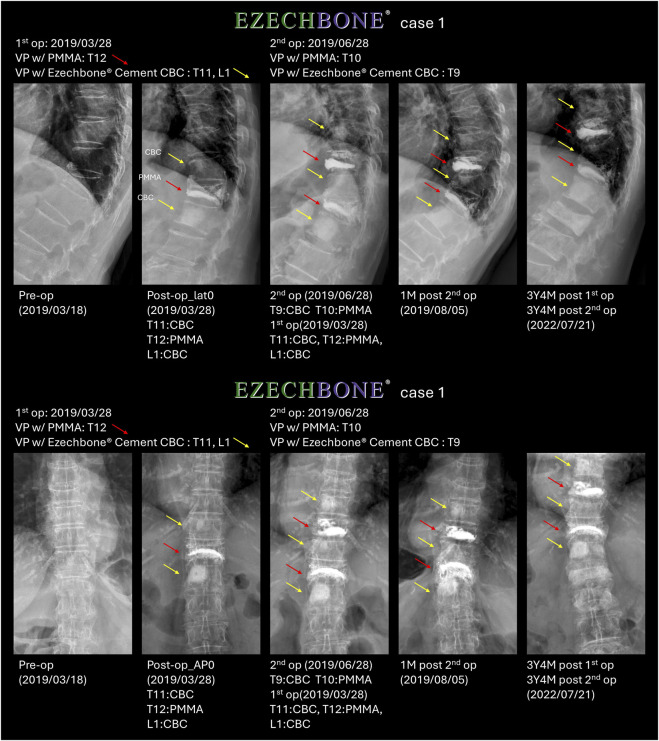
The preoperative and a series of follow-up radiographs in case 1.

This 80-y/o female patient had multiple myeloma status post-chemotherapy complicated with biochemical relapse, hypercalcemic incidents, and Bortezomib-related peripheral sensory/motor neuropathy. Other underlying diseases included hypertension, congestive heart failure, hypothyroidism secondary to autoimmune thyroiditis, right femoral neck fracture status post-bipolar hemiarthroplasty, and left distal radius fracture status post k-wire fixation. She suffered from progressive back pain without any trauma incident. After failure from conservative treatment, T12 vertebroplasty with PMMA cement accompanied with vertebroplasty of adjacent segments (T11 and L1) with Ezechbone® Cement CBC-400 was performed. The T-score measured with DXA was −3.4, and an anabolic agent, teriparatide 20 mcg, was administered every day. The back pain resolved initially but recurred within 2.5 months. T10 progressive collapse was found and turned out to be fresh fracture on magnetic resonance imaging (MRI). The second operation took place 3 months after index operation, and the procedures included T10 vertebroplasty with PMMA and T9 vertebroplasty with CBC-400. In Figure 9, the 3-year followed up radiographs after the second operation indicated that the resorbed CBC-400 in T9, T11, and L1 bodies was replaced with denser autologous bone, and the vertebral body heights of T9, T11 and L1 were well maintained.

#### 3.2.2 Case 2

**FIGURE 10:**
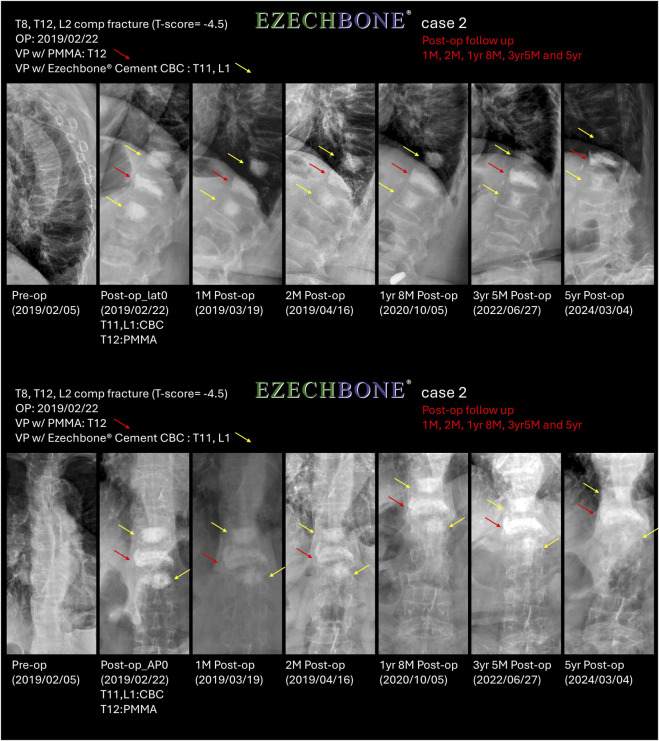
The preoperative and a series of follow-up radiographs in case 2.

This 75-y/o female patient had underlying diseases of end-stage renal disease under regular hemodialysis complicated with secondary hyperparathyroidism, bladder transitional cell carcinoma status post-transurethral resection of bladder tumor and chemotherapy, coronary artery disease status post-angioplasty with stents placed in two vessels, hypertension, and dyslipidemia. She presented to our emergency department due to falling down from a scooter, which disabled her from arising from the bed on her own. Old T8 and fresh T12 compression fractures were noted on MRI. After conservative treatment failed, she received T12 vertebroplasty with PMMA cement and vertebroplasty of adjacent segments (T11 and L1) with Ezechbone® Cement CBC-400. Postoperatively, she was relieved from the severe back pain, and her daily activity was restored. The BMD measurement revealed the T-score as low as −4.5. An anti-resorptive agent, denosumab 60 mg, was administered every 6 months. Some falling-down incidents had occurred during the follow-up period and caused L2 and L4 compression fractures, which were successfully managed conservatively. In Figure 10, the 5-year followed-up radiographs showed that the resorbed CBC-400 in T11 and L1 bodies was substituted with denser autologous bone, and the vertebral body heights of T11 and L1 were well maintained.

#### 3.2.3 Case 3

**FIGURE 11:**
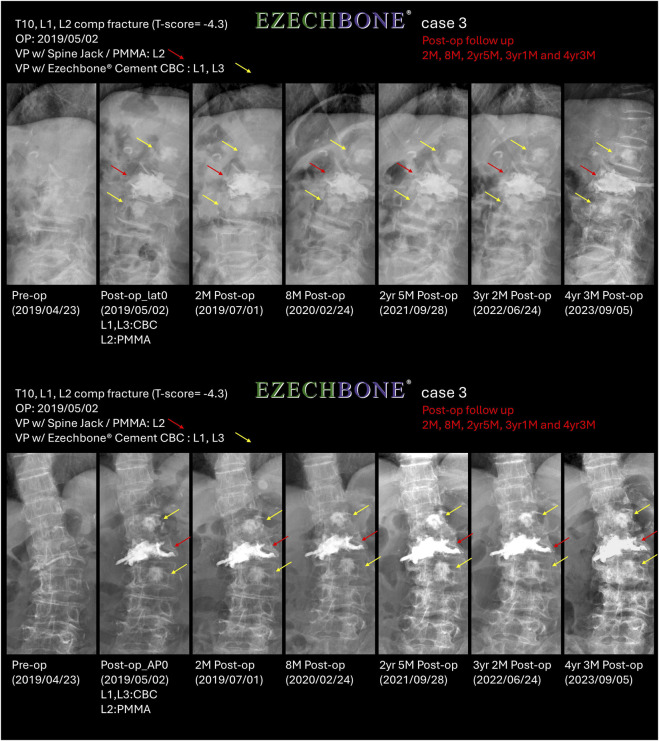
The preoperative and a series of follow-up radiographs in case 3.

This 88-y/o female patient with hypertension had a sudden onset of back pain while walking. Progressive back pain developed to debilitation under conservative treatment. One shot of denosumab 60 mg had been administered within 2 months, but L2 compression fracture progressed to severe body height loss. L2 vertebroplasty with SpineJack and PMMA was combined with L1 and L3 vertebroplasty with Ezechbone® Cement CBC-400. BMD examination showed the T-score to be −4.3, and denosumab therapy was replaced with teriparatide therapy. In Figure 11, the 4.3-year followed-up radiographs revealed that the resorbed CBC-400 in L1 and L3 bodies was substituted with denser autologous bone, and the vertebral body heights of L1 and L3 were well maintained.

#### 3.2.4 Case 4

**FIGURE 12:**
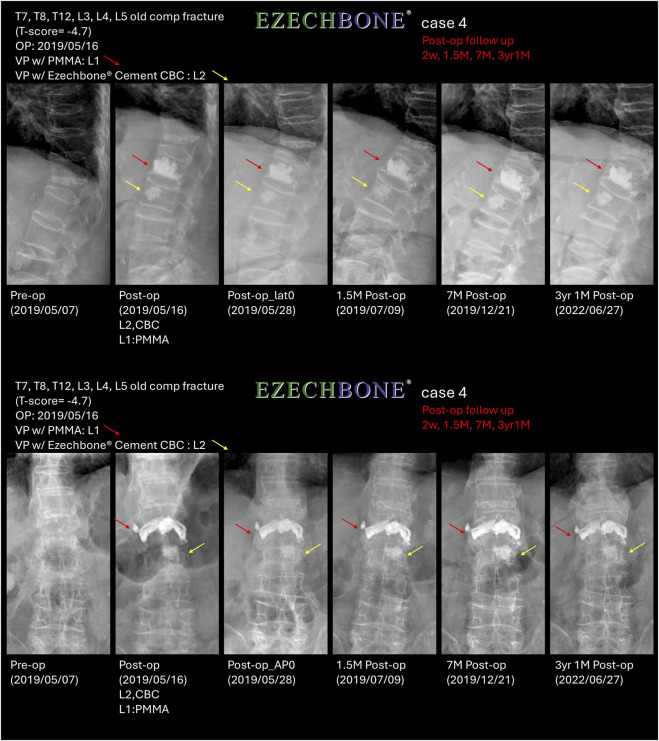
The preoperative and a series of follow-up radiographs in case 4.

This 83-y/o female patient had chronic back pain with severe accentuation for 2 weeks. No recent trauma incident was reported. Old T8 and T12 compression fractures were noted on X ray, while MRI revealed fresh L1 compression fracture. The pain was relieved after L1 vertebroplasty with PMMA and L2 vertebroplasty with Ezechbone® Cement CBC-400 were performed. BMD examination revealed the T-score to be −4.7. Teriparatide therapy started perioperatively. In Figure 12, the 3-year followed-up radiographs revealed that the resorbed CBC-400 in the L2 body was substituted with denser autologous bone, and the vertebral body height of L2 was well maintained. In comparison, T8 and T12 had a little further collapse.

## 4 Discussion

Due to their similar biochemical and histopathologic features, the OVX-induced osteoporotic goat model has long been used to assess the healing potential of several bone graft materials in humans (Leung et al., 2001; Leung et al., 2006; Li M. et al., 2010; Cao et al., 2012; Yu et al., 2015). The present OVX procedure, accompanied by a low-calcium diet and breeding away from light according to the referenced protocols, successfully induced osteoporosis in the experimental female goats, as confirmed by the much more porous and thinner trabecular microstructure observed in the OVX goat than in the healthy control goat.

In an early study, Kubo et al. (1999) observed clusters of osteoclasts on the osteoclast-etched bone surface in OVX goats. Such phenomenon was also observed in the present OVX goat spine (labeled by arrows in Figure 2c,d) (Demontiero et al., 2012). The significant reductions in trabecular thickness, trabecular number, and bone coverage in Figure 2 were also observed in the trabeculae of lumbar vertebral bodies of OVX goats at 24 months post-OVX (Yu et al., 2015). In the present study, we observed that the trabecular structure of OVX goat was much more porous and the trabeculae therein were much thinner than those of the healthy control goat. The newly developed trabecular bone network in the OVX_CBC goat was much denser than that in the osteoporotic OVX goat. It is also interesting to notice that the newly developed trabeculae near the CBC-400 cement were much smaller-scaled and more randomly oriented than those away from the cement and those in the healthy control goat (Figure 3a,b). This indicates that Ezechbone® Cement CBC-400 has an excellent bone-regenerating efficacy when implanted in osteoporotic goat spine.

Quite a few reports suggested that calcium phosphate could induce osteogenic differentiation of mesenchymal stem cells (MSCs) and facilitate the formation of new bone in bony defects (Müller et al., 2008; Song et al., 2013). Chang et al. (2000) suggested that calcium phosphate implant-induced elevation of the extracellular calcium ion concentration might play an important role in the osteogenic differentiation process. Among the studies on the effect of calcium sulfate on bone healing, Aquino-Martínez et al. (2017) reported that calcium sulfate could enhance MSC migration and recruit osteoprogenitor cells. Ricci et al. (2005) reported that, when dissolved in body fluid, the calcium ions released from sulfate formed a layer of calcium phosphate attaching to the adjacent new bone. These findings may partly explain the observed excellent healing efficacy of the present Ca/P/S-derived CBC-400 cement in the osteoporotic goat.

It was reported that, during a calcium-based implant-involved bone remodeling process, the implant and woven bones were gradually replaced by the lamellar bone. This process generally takes a relatively long period of time (Barrère et al., 2006). Woven bones are commonly present during skeleton development or in fracture callus. Lamellar bones, in contrast, are usually composed of stronger and better-aligned collagen fibers. The newly formed bone in the present CBC-400-implanted OVX_CBC goat was primarily lamellar bone aligned parallel to the implanted cement surface. In this OVX_CBC goat, the absence of the woven bone was probably due to the CBC-400-facilitated fast bone healing process. Compared to the OVX goat, the lamellar bone morphology in the OVX_CBC goat appeared much more randomly oriented. This is probably due to the inherent randomly oriented nature of the inorganic CBC-400 cement and the fast new bone deposition onto and near the cement, which, in turn, caused the newly developed lamellar bone to become randomly oriented. The new bone network developed within the implantation site in the OVX_CBC goat at 24W post-implantation suggests that the implanted CBC-400 cement could help the skeleton bridge the gap of bone void by enhancing the healing and new bone formation processes. The microcracks within the implanted cement could effectively provide a shortcut for new bone ingrowth, thereby accelerating the new bone formation process.

Most studies on the healing effect of bone grafts/implants in osteoporotic bone compared their results only to the osteoporotic control group or sham (surgically created bony void without implantation) in small- or medium-sized osteoporotic animals. They did not compare to normal healthy blank control (Leung et al., 2006; Cao et al., 2012). This information is quite important because a healthy baseline is essential to determine whether the graft-induced healing can fully restore the bone to its original, non-osteoporotic state. Furthermore, studies on this subject using large animal models, such as osteoporotic goats, are even much more lacking. Blouin et al. (2006) found that, at 3 months after implantation of a β-tricalcium phosphate/hydroxyapatite (HA)/hydroxypropyl methylcellulose composite graft in osteopenic rats, the regenerated bone still showed a more porous morphology than the healthy native bone, with approximately a one-third decrease in trabecular bone volume and bone numbers. Inferior bone healing in HA-implanted osteoporotic rats to that in the non-osteoporotic control was also reported by Do Prado Ribeiro et al. (2012). The amount of HA-induced new bone in osteoporotic bone was only 34% of the sham group. A similar phenomenon was observed even in autograft (the so-called “gold standard”)-implanted osteoporotic bone. Implanting autografts in the femur of normal and osteoporotic rabbits, Li J. P. et al. (2010) reported that the area of new bone in cancellous bone in osteoporotic rabbits was only approximately half of that in the normal rabbits. Xiao et al. (2007) suggested that the osteogenic differentiation of MSCs could be altered in osteoporotic bone, and, in turn, could change the fracture repair process in osteoporotic bone (Lu et al., 2005). Durão et al. (2014) investigated bone graft-mediated healing of bone defects in Bio-oss® (a bovine bone) and observed impaired differentiation and osteoblastic gene expression. This could probably explain impaired quality of bone fracture healing in osteoporotic animals. These findings indicate that re-establishing normal bone repair in osteoporotic bone remains to be a great challenge. Osteoporosis was reported to delay the bone remodeling process, leave more calluses or woven bones in the fracture region, and delay the new bone maturation process to recover various desired properties (Walsh et al., 1997; Kubo et al., 1999; Meyer Jr et al., 2001; Namkung-Matthai et al., 2001; Lill et al., 2003; McCann et al., 2008).

To improve the impaired osteogenic ability of calcium phosphate bone cements (CPCs), the majority of researchers have focused on incorporating drugs or trace elements into CPCs to enhance their bone-healing ability. For example, by adding alendronate into a CPC/PLGA composite, van Houdt C. I. A. et al. (2018) successfully stimulated more new bone ingrowth than that without alendronate. Lu et al. (2022) investigated the bone stimulation ability of lithium (Li)-doped CPC and found that the Li-doped CPC had favorable osteogenesis and osteointegration capabilities compared to pure CPC. Thormann et al. (2013) compared the healing ability between CPC and strontium (Sr)-doped CPC (SrCPC) in ovariectomised (OVX) rats. Their results showed a significantly higher bone formation rate in the SrCPC group than in the CPC group at both the defect zone and the tissue–implant interface.

Another approach to improving the relatively low biodegradation rate of conventional CPCs is by adding porous agents to CPCs. In a recent study, Kang et al. (2025) added alpha-ketoglutarate polyester microspheres into CPCs to enhance their biodegradability, osteogenic differentiation potential, and biomineralization. With this formula, the authors observed an enhanced bone growth rate compared to pure CPCs in an osteoporotic SD rat model. With a different approach, in the present study, an inorganic fast-degrading CSC is incorporated into an in-house fabricated CPC as a bone-healing stimulant, which is confirmed from the present animal study and preliminary clinical results to carry a high potential for achieving a timely healing in highly osteoporotic bone defects.

Due to its delicately designed chemistry and structure in Ezechbone® Cement CBC-400, the new bone morphology in the CBC-400-implanted OVX_CBC goat was comparable to that in the normal control goat in approximately 6 months after implantation. No sign of osteoporosis-related delay was found, such as leaving more calluses or woven bones in the fracture region and in the bone maturing process. The quick and effective recovery of trabecular architecture observed in the OVX_CBC goat indicates that, even without use of anti-osteoporosis drug or growth factor, the present Ca/P/S-based CBC-400 cement exhibits a unique potential to heal osteoporotic fractures.

The encouraging results in the animal study have been further confirmed by the 10 clinical vertebral compression fracture cases. Even at an average age of almost 80 years, with half of the patients having prior osteoporotic vertebral fractures and all with multiple comorbidities, none of these patients had ever encountered any new fractures in the adjacent vertebrae filled with Ezechbone® Cement CBC-400. This clinical result demonstrates that prophylactically strengthening adjacent osteoporotic vertebrae with Ezechbone® Cement CBC-400, in combination with conventional PMMA vertebroplasty for OVCF, can effectively reduce the risk of future adjacent vertebral fractures. As a comparison, the institutional historical data were reviewed, and it was found that, among 244 patients who underwent conventional vertebral PMMA cement augmentation between 6 September 2014 and 31 March 2023, 21 (8.6%) had developed adjacent fractures within 3 years. Given the pilot nature of the study with a relatively small sample size, the clinical data from the present study should be interpreted with prudence. Further larger-scale, randomized controlled trials are warranted to confirm the long-term safety and clinical efficacy of this cement in a broader patient population.

As a final remark, when implanted, the CBC-400 cement is nondispersive and safely confined within the surgically created bone cavity in the osteoporotic goat’s vertebral body. This nondispersive and self-confining feature is expected to largely reduce the risks of cement leakage and cement embolism.

## 5 Conclusion

1. The present OVX procedure, accompanied with a low-calcium diet and breeding away from light, successfully induced osteoporosis in female experimental goats.
2. When implanted, the Ca/P/S-based Ezechbone® Cement CBC-400 was nondispersive and safely confined within the surgically created bone cavity of the osteoporotic goat’s vertebral body. This nondispersive and self-confining feature is expected to largely reduce the risks of cement leakage and cement embolism.
3. Histological results showed that a new bone network developed within the cement, and the numerous cement residues were intimately integrated with the surrounding new bone. Between CBC-400 cement and the host bone, a tight interface was consistently observed, without interposition of any fibrous tissues.
4. The trabecular structure in the OVX goat was much more porous and thinner than that in the healthy control goat. The newly formed trabecular network in the OVX_CBC goat was much denser than that in the osteoporotic OVX goat and was comparable to that in the healthy control goat. Under polarized light, a much more randomly oriented, smaller-sized, and more vibrant lamellar bone morphology was observed in the OVX_CBC goat than in the OVX goat, indicating an excellent bone-regenerating efficacy of CBC-400 cement. These results suggest that, even without use of an anti-osteoporosis drug or growth factor, the present Ca/P/S-based CBC-400 cement graft material has a unique potential to heal osteoporotic voids or fractures.
5. The results of 10 consecutive vertebral compression fracture cases showed that, even at an average age of almost 80 years, with half of the patients having prior osteoporotic vertebral fractures and all with multiple comorbidities, none of these patients had ever encountered any new fractures in the adjacent vertebrae augmented with Ezechbone® Cement CBC-400. Prophylactically augmenting adjacent osteoporotic vertebrae with CBC-400 cement, in combination with conventional PMMA vertebroplasty for OVCF, has demonstrated a high potential for effectively reducing the risk of future adjacent vertebral fractures. Given the pilot nature of the study with a relatively small sample size, the clinical data from the present study should be interpreted with prudence. Further larger-scale, randomized controlled trials are warranted to confirm the long-term safety and clinical efficacy of this cement in a broader patient population.

## Data Availability

The datasets presented in this article are not readily available because the datasets for this article are not publicly available due to concerns regarding participant/patient anonymity. Requests to access the datasets should be directed to Jiin-Huey Chern Lin, jhchernlin.jmd@gmail.com.
