# Supplementary material for: Study of a novel Ca/P/S-based cement implanted in the osteoporotic goat spine and its clinical application in preventing adjacent vertebral fractures
Source: Front Bioeng Biotechnol. 2026 May 29;14:1784212. doi: 10.3389/fbioe.2026.1784212 (PMC13260643; doi:10.3389/fbioe.2026.1784212)
Supplement: Supplementary file 1 [file DataSheet1.pdf]

**Supplementary table**

Supplementary table summarizing key material properties of Ezechbone® Cement CBC-400 (Chen et al., 2014)

| | |
|---|---|
| Ca/P/S weight ratio | 56.07/18.23/25.70 |
| Working time (min) | 10.1±0.9 |
| Setting time (min) | 11.1±1.2 |
| Compressive strength immersed in Hank's solution (MPa) | (1d) 17.9±1.6<br>(14d) 13.6±2.1 |
| Porosity immersed in Hank's solution (%) | (1d) 37.5±2.0<br>(14d) 45.3±0.7 |
| Weight loss immersed in Hank's solution (%) | (1d) 18.6±1.0<br>(14d) 26.3±1.0 |
